# Light and CO_2_ Modulate the Accumulation and Localization of Phenolic Compounds in Barley Leaves

**DOI:** 10.3390/antiox10030385

**Published:** 2021-03-05

**Authors:** Lena Hunt, Karel Klem, Zuzana Lhotáková, Stanislav Vosolsobě, Michal Oravec, Otmar Urban, Vladimír Špunda, Jana Albrechtová

**Affiliations:** 1 Department of Experimental Plant Biology, Faculty of Science, Charles University, Viničná 5, 12844 Praha, Czech Republic; huntl@natur.cuni.cz (L.H.); zuzana.lhotakova@natur.cuni.cz (Z.L.); vosolsob@natur.cuni.cz (S.V.); 2 Global Change Research Institute, Czech Academy of Sciences, Bělidla 4a, 60300 Brno, Czech Republic; klem.k@czechglobe.cz (K.K.); oravec.m@czechglobe.cz (M.O.); urban.o@czechglobe.cz (O.U.); Vladimir.Spunda@osu.cz (V.Š.); 3 Department of Physics, Faculty of Science, University of Ostrava, Dvořákova 7, 70103 Ostrava, Czech Republic

**Keywords:** phenolic compounds, histochemical localization, elevated CO_2_, image analysis, irradiance, plant stress, hydroxycinnamic acids, hydroxybenzoic acids, flavonoids, barley

## Abstract

Barley (*Hordeum vulgare*) accumulates phenolic compounds (PhCs), which play a key role in plant defense against environmental stressors as antioxidants or UV screening compounds. The influence of light and atmospheric CO_2_ concentration ([CO_2_]) on the accumulation and localization of PhCs in barley leaves was examined for two varieties with different tolerances to oxidative stress. PhC localization was visualized in vivo using fluorescence microscopy. Close relationships were found between fluorescence-determined localization of PhCs in barley leaves and PhC content estimated using liquid chromatography coupled with mass spectroscopy detection. Light intensity had the strongest effect on the accumulation of PhCs, but the total PhC content was similar at elevated [CO_2_], minimizing the differences between high and low light. PhCs localized preferentially near the surfaces of leaves, but under low light, an increasing allocation of PhCs in deeper mesophyll layers was observed. The PhC profile was significantly different between barley varieties. The relatively tolerant variety accumulated significantly more hydroxycinnamic acids, indicating that these PhCs may play a more prominent role in oxidative stress prevention. Our research presents novel evidence that [CO_2_] modulates the accumulation of PhCs in barley leaves. Mesophyll cells, rather than epidermal cells, were most responsive to environmental stimuli in terms of PhC accumulation.

## 1. Introduction

Barley (*Hordeum vulgare*) is one of the most widely cultivated and economically important crops. It is considered a founder crop in agriculture, with archaeological evidence placing its domestication back 10,000 years ago [1]. Today, it is the fourth most produced grain in the world, behind only corn, wheat, and rice [2]. The primary use of barley has been as livestock feed, and in the production of beer and whiskey. However, high levels of antioxidants, vitamins, minerals, and essential amino acids in young barley leaves, as well as beta-glucans in barley grains, have led to the profitable marketing of barley as a health food as well [3,4]. It is estimated that by 2050, barley production will need to increase 54% over levels in the year 2000 to keep up with the demand of a rising global population [5]. Due to its global economic importance, there is much interest in how the production of barley will fare with ongoing climate change. Areas of the world seeing increased frequencies of droughts and heatwaves will experience significant drops in barley yield [6]. The Mediterranean basin may see yield decreases up to 25% [6], while Central Europe can expect greater variability between harvests [7]. Despite these projections, barley maintains a reputation for being resilient to environmental stress and is expected to be less impacted than other crops, such as corn or potatoes [7].

The tolerance to abiotic stressors is considerably influenced by plant secondary metabolites. Barley is rich in phenolic compounds (PhCs), a large family of compounds including phenolic acids, flavonoids, lignins, and tannins, which provide protective functions in planta [8]. PhCs have been documented to increase barley tolerance to oxidative stress—a state of excess reactive oxygen species (ROS) induced by a range of environmental stressors. ROS play a dual role: functioning as signaling molecules that regulate cellular metabolism and defense response systems in low concentrations—but causing detrimental oxidation of proteins, lipids, and nucleic acids, leading to cell damage or death when they occur in excess [9,10]. Irradiance induces ROS accumulation in plants, both directly and indirectly; directly, excess irradiance can result in an over-reduced NADP^+^ pool, leading to the reduction of molecular oxygen and, thus, the formation of ROS during photosynthesis. Indirectly, abiotic stress typically reduces photosynthetic capacity–previously optimum light conditions can become excessive and induce photooxidation [11]. PhCs have shown a protective role in barley against a number of oxidative stress inducing factors, including excessive irradiation [12], drought and salinity [13], pathogens [14], air pollution [15], and heavy metal exposure [16].

The protective function of PhCs is a result of their UV (ultraviolet) screening and antioxidant activity [17,18]. PhCs that attenuate UV irradiation prevent the direct generation of ROS and subsequent oxidative stress, while PhCs with antioxidant activity scavenge ROS. Chemical structure plays a role in PhC function [19,20]. For instance, compounds, such as hydroxycinnamic acids, are more effective at UV screening, while flavonoids with a dihydroxylated B-ring possess higher antioxidant activity [21]. There is some debate about the role of flavonoids as antioxidants in planta [22], however, the exclusive role of PhCs in UV screening seems unlikely since UV is not necessary to induce their accumulation, and the synthesis of antioxidant flavonoids increases at the expense of hydroxycinnamic acids that are more efficient in UV screening [23]. Antioxidative PhC biosynthesis is more likely tied to ROS signals alongside the over-reduction of the photosynthetic electron transport chain [24], which would explain their accompaniment to various sources of environmental stress. PhCs accumulating in epidermal cell walls and vacuoles may play a more significant role as UV screeners in photoprotection [25,26]. However, PhCs, particularly with a dihydroxylated B-ring that possess high antioxidant activity, have been shown to accumulate deeper in the leaf–in mesophyll vacuoles and chloroplasts [22,27,28]. A third and somewhat underrated function of PhCs (specifically flavonoids) is their possible role as an energy escape valve, utilizing ATP and NADPH and promoting phosphate cycling between the cytosol and chloroplast [22]. This function may be especially relevant in elevated [CO_2_] conditions, where an abundance of CO_2_ results in an increase in non-structural carbohydrate biosynthesis [29]. Although PhC accumulation is known to be affected by a plant’s environment, few methods exist to visually measure the localization of phenolic compounds within leaf tissue, which is an important prerequisite for understanding their function in planta.

This study investigates the combined effects of [CO_2_] and light intensity on the accumulation and localization of PhCs in two varieties of barley with different tolerances to oxidative stress. We expected that the variety Bojos would accumulate more PhCs with antioxidant activity in mesophyll cells, in contrast to the oxidative-stress sensitive variety Barke. We hypothesized that: (1) both elevated [CO_2_] and high light intensity would increase the total accumulation of PhCs, and that light intensity especially would change the distribution of PhCs within leaf cross-sections to comply with UV screening and antioxidant roles; and (2) relative tolerance or sensitivity of barley cultivars could be connected to differences in PhC accumulation, composition, and/or localization. Histochemistry, fluorescence microscopy, and image analysis were combined to provide a unique method for analyzing PhCs visually, alongside targeted high-performance liquid chromatography/high-resolution mass spectrometry (HPLC-HRMS) analysis.

## 2. Materials and Methods

### 2.1. Plant Material and Growth Conditions

The two barley varieties investigated in this study were selected for their widespread cultivation in Europe and their different tolerances to oxidative stress. The Barke variety is reportedly sensitive to photooxidative stress: it is known to develop necrotic physiological leaf spots when exposed to excess light [30] and show pronounced reductions in photosynthetic activity when exposed to excess UV [31]. By contrast, Bojos is one of the most widely grown barley varieties in the Czech Republic [32,33] and rarely shows even mild symptoms of photooxidative stress, according to the multi-annual testing of Central Inspection and Testing Institute of Agriculture for the Czech Republic [34]. We refer to these varieties as oxidative stress sensitive (Barke) and ‘relatively tolerant’ (Bojos).

Barley plants of Barke and Bojos varieties were grown in six growth chambers (FS-SI-3400, Photon System Instruments, Drásov, CZ). Cultivation occurred over four weeks under three different [CO_2_] treatments: low [CO_2_]—200 ppm (LC), ambient [CO_2_]—400 ppm (AC), and elevated [CO_2_]—700 ppm (EC) and two light regimes: low light (LL) with photosynthetically active radiations (PAR) and UV-A maxima of 400 µmol m*^−^*^2^ s*^−^*^1^ and 0.75 W m*^−^*^2^, respectively and high light (HL) with PAR and UV-A maxima 1500 µmol m^−2^ s^−1^ and 4 W m*^−^*^2^, respectively (UV-A lamps LT 30W T8/010UV with maximum emission at 370 nm, Narva Lichtquellen, Brand-Erbisdorf, Germany). The light intensity, temperature, and air humidity changed gradually from night values to a daytime maximum between 5:00 and 10:00, then remained constant between 10:00 and 15:00, and finally changed again to nighttime values between 15:00 and 20:00 to simulate natural rhythms with 15 h day and 9 h night (see Figure S1). Daily integrals for PAR were 14.4 and 54 mol m^−2^ day^−1^ and for UV-A 27 and 144 J m^−2^ day^−1^ under LL and HL, respectively. The air temperature varied between 15–25 °C and relative air humidity between 90–60%, for night and day respectively (see Figure S1). Light treatments were selected to mimic realistic Central European light conditions in spring for cloudy-day field conditions (LL) and sunny conditions slightly exceeding the light saturation point for barley (HL) [35].

Five plants were grown in each pot of size 11 × 11 × 12 cm filled with fertilized peat substrate TS2 (Klasmann-Deilmann, Geeste, Germany). Each combination of variety, [CO_2_], and light intensity was replicated 6 times (6 pots containing 5 plants each). To avoid potential artefacts from individual growth chamber, the pots were transferred every seven days between growth chambers maintaining the same [CO_2_] and light treatment. Positions of pots within growth chambers were also randomized. At the end of four weeks, when the plants reached the growth stage of 6 leaves (DC 16), samples for histochemical analysis, HPLC-HRMS, and measurements of chlorophyll index and UV screening of chlorophyll fluorescence (UV screening index) were taken.

### 2.2. Histochemical Detection of Phenolic Compounds

For all histochemical analyses, leaf cross-sections and transverse-sections were made from the third leaf from the top, representing the youngest completely developed leaf, in the middle of the leaf. Cross-sections were made at approximately 85 µm thickness using a hand microtome (Leica RM 2255, Wetzler, Germany). Paradermal images were created by slicing a whole leaf into 1 mm squares before treatment with reagents (Figure S2), which allowed better infiltration of reagents into epidermal cells via adjacent mesophyll cells while keeping the epidermal cells intact.

Initially, several histochemical tests were performed to confirm the presence of various PhCs. With light microscopy, phloroglucinol-HCl was used to detect the presence of lignin [36], and Vanillin-HCl was used to detect condensed tannins [37]. With fluorescence microscopy, Naturstoff reagent A was used to detect flavonoids [38]. Naturstoff reagent A was determined to provide the most data about induced PhC accumulation, and so we selected it for continued use with experimental samples. The samples prepared for use with Naturstoff reagent A were mounted in a 100 mM KPi pH 6.8, 1% NaCl (*w/v*) buffer solution [24]. A few drops of 0.1% (*w/v*) Naturstoff reagent A were transferred under the coverslip using filter paper approximately five minutes before using the microscope. Naturstoff reagent A forms chelates with characteristic fluorescence depending on the hydroxyl group substitutions on the B and C rings [38]. For this study, we processed three samples treated with Naturstoff reagent A from different leaves per replication. To process and photograph samples in a short enough period to not introduce additional error factors, such as sample drying or loss of fluorescent signal, we selected to use standard fluorescence microscopy. This also enabled us to readily compare images of leaf cross-sections under white light, blue light, and UV radiation (see Microscope image acquisition for specifications). This is beneficial as the autofluorescence of certain PhCs change depending on the wavelength of light they are excited by. For example, flavonoids fluoresce yellow under both UV and blue light excitation, caffeic esters fluoresce blue-white under UV radiation and yellow under blue light excitation, and ferulic acid fluoresces blue under UV radiation, but has no fluorescence under blue light excitation [39,40].

#### 2.2.1. Microscopic Image Acquisition

The samples prepared for histochemical detection tests were photographed using the microscope AX70 (Olympus, Hamburg, Germany) at 40× magnification (to capture the whole leaf) and 100× magnification (to capture details on tissue and cellular localization) under bright field for phloroglucinol-HCl and Vanillin-HCl, and using fluorescence microscopy under both UV and blue light excitation with Naturstoff reagent A. Excitation was achieved using epi-fluorescent filter cubes: wide UV (U-MWU, excitation 330–385 nm, dichroic cut-off 400 nm, emissions > 420 nm) and wide blue (U-MWB, excitation 450–480 nm, dichroic cut-off 500 nm, emission > 515 nm) (Olympus, Hamburg, Germany). Image analysis for quantitative analysis on histochemical localization was performed on images of cross-sections treated with Naturstoff reagent A and excited with blue light.

#### 2.2.2. Quantitative Analysis of Phenolic Compounds Using Image Analysis

**Figure 1:**
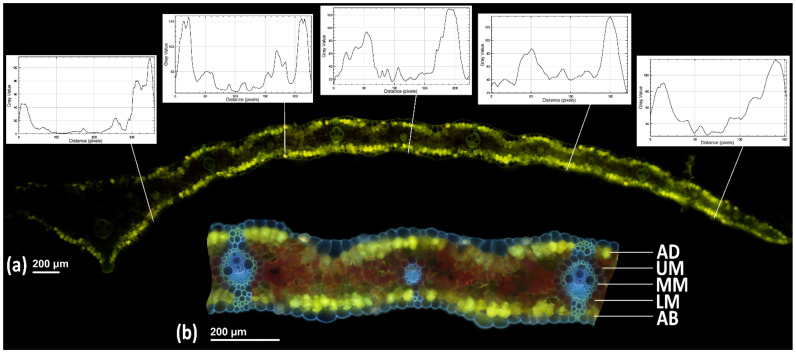
Method for quantitative measurements of relative intensities of PhC fluorescence throughout the mesophyll tissue inside half of a *Hordeum vulgare* leaf lamina using LinSys Cycloides and Plot Profile function in ImageJ. The plugin LinSys Cycloides automatically generates equally spaced lines over the image to provide systematic random sampling sites. (**a**) Straight line and Plot Profile tools are used to measure the intensity of pixel brightness at the sampling sites throughout the image of leaf PhC fluorescence (5 white graphs). (**b**) Pixel values of sample lines are divided into 5 segmental sections corresponding to 5 mesophyll depths: adaxial layer of mesophyll adjacent adaxial epidermis (AD), upper mesophyll (UM), middle mesophyll (MM), lower mesophyll (LM), abaxial layer of mesophyll adjacent to abaxial epidermis (AB).

The reaction with Naturstoff reagent A was mainly detected in mesophyll cells and displayed a gradient across internal cell depths. To obtain a relative measure of PhC fluorescence localization, the chlorophyll autofluorescence was digitally removed using a macro in Adobe Photoshop and applied to all photographs as a batch. The macro selected all yellow color and moved it to a new layer in Photoshop, showing only the PhC fluorescence. The PhC fluorescence-only images were opened in ImageJ. The plug-in LinSys Cycloides was used to randomly generate equally spaced vertical lines to provide unbiased systematic random sampling to achieve unbiased estimations of histochemical localization across a gradient in the mesophyll [41] while not intersecting vascular tissues. The distance between these lines was adjusted so that each leaf was vertically intersected in five equally-spaced locations—the first being near the midrib and the fifth being near to the edge of the leaf. The straight-line tool and Plot Profile function provided intensity data for each pixel intersected by the sampling line (Figure 1). The data from the five intersections through the mesophyll tissue along the width of the leaf were further divided by mesophyll depth: adaxial mesophyll layer (AD), upper mesophyll (UM), middle mesophyll (MM), lower mesophyll (LM), and abaxial mesophyll layer (AB). The data from three samples per replication and five locations within leaf width were averaged and used for subsequent statistical analyses.

### 2.3. Targeted HPLC-HRMS Analysis of Contents of Phenolic Compounds

The third leaf from the top from each replication was sampled between 11:00 and 14:00 (Central European Time) and immediately after scanning for leaf area frozen in liquid nitrogen for target high-performance liquid chromatography analyses (i.e., the leaves from the same position on the plant were sampled for HPLC and histochemical analyses). The samples were homogenized using a mortar and pestle with liquid nitrogen and then extracted using methanol:chloroform: H_2_O solution (v:v:v, 1:2:2). An aliquot of the upper (polar) phase was used to analyze metabolites using an UltiMate 3000 high-performance liquid chromatography (HPLC) (Thermo Fisher Scientific, US/Dionex RSLC, Dionex, Waltham, MA, USA) coupled with an LTQ Orbitrap XL high-resolution mass spectrometer (HRMS) (Thermo Fisher Scientific, Waltham, MA, USA) that was equipped with a heated electrospray ionization source. All samples were analyzed in the positive and negative polarity of Orbitrap, operated in full-scan mode over a range of m/z 50 to 1000 (positive mode) and 65 to 1000 (negative mode). For details see [12].

### 2.4. Chlorophyll and UV Screening Indices

A Dualex optical sensor was used to measure chlorophyll and UV screening indices (Dualex Flav, Force A, Orsay, FR). Measurements are in Dualex units based on light transmittance and UV screening of chlorophyll fluorescence excitation. The UV screening index measurement is mainly related to the content of ortho-dihydroxylated flavonoids, which have absorption maxima around 375 nm (the excitation wavelength of the Dualex instrument) [42]. The UV screening is often referred to as “epidermal”, however the screening functions can come also from the layer below the epidermis. For more information on Dualex, see [43]. Three measurements in the central part of the same leaf (third leaf from the top) used for targeted HPLC-HRMS and histochemical analyses were performed. The means from one leaf were used for statistical analyses (6 replicates).

### 2.5. Statistical Analysis

Data were analyzed using a four- or three-way fixed-effect ANOVA model using Statistica 12 software (StatSoft, Tulsa, OK, USA). Fishers’ LSD post hoc test (*p* = 0.05) was used to identify significant differences between means. The bar graphs with indicated standard errors and the multiple scatter graphs with linear regressions were created in the program SigmaPlot 11.0 (Systat Software, San Jose, CA, USA). The redundancy analysis (RDA) and biplot of RDA results were set up in the software CANOCO 5 [44].

## 3. Results

### 3.1. Localization of Phenolic Compounds within Leaf Cross-Sections

**Figure 2:**
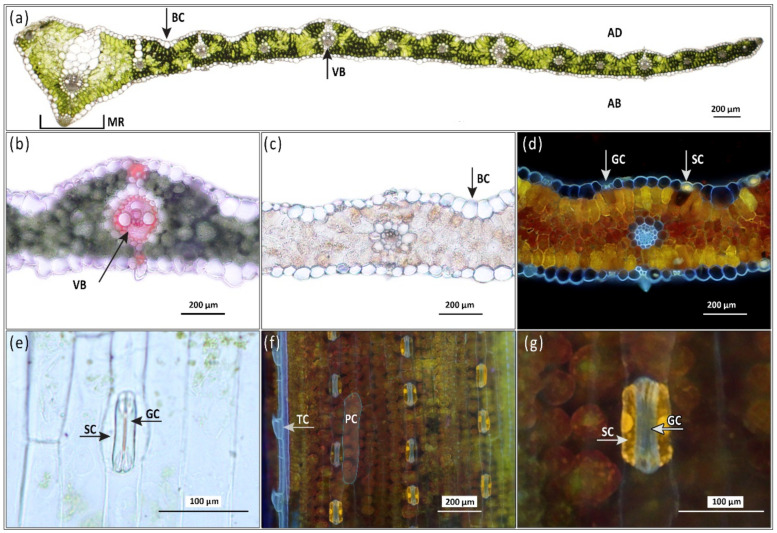
Examples of histochemical analyses of barley (*Hordeum vulgare)* leaves. (**a**) Partial barley leaf cross section from one half of the lamina including midrib (MR) on left, bulliform cells (BC) of the abaxial epidermis, adaxial side (AD), and abaxial side (AB). Fresh hand section not treated histochemically, imaged at 40× magnification, bright field. (**b**) Phloroglucinol-HCl test for lignins (pink-red color) in vascular bundle (VB) and sclerenchymatous extensions of bundle sheath leading to epidermal cells, 100×, bright field. (**c**) Vanillin HCl test for condensed tannins (bright red) not detected in mesophyll, 100×, bright field. (**d**) Naturstoff reagent A test (yellow fluorescence) for flavonoids, detected in the mesophyll but absent in the epidermis except for stomatal subsidiary cells, arrows point to stomatal guard cells (GC) and subsidiary cells (SC), 100×, UV fluorescence. (**e**) Barley stomata from an epidermal peel showing guard cells (GC) and subsidiary cells (SC), 200×, bright field. (**f**) The paradermal view on a segment of a barley leaf treated with Naturstoff reagent A showing vacuolar flavonoids (yellow fluorescence). Leaf segments were of 1 mm^2^ size so the reagent could penetrate the intact epidermal cells via mesophyll cells. Flavonoids are present only in SC and GC and in the mesophyll cells below the transparent epidermal pavement cells. Trichome cells (TC) along leaf vein show blue autofluorescence and pavement cells (PC) (highlighted to indicate size and position) lack fluorescence except a faint blue color along cell walls, 100×, UV fluorescence. (**g**) Zoomed in view of stomatal guard cells (GC) and subsidiary cells (SC) treated with Naturstoff reagent A showing intracellular fluorescence of phenolic compounds. Adjacent epidermal pavement cells do not show flavonoid fluorescence and their transparency enables the viewing of flavonoid fluorescence in the mesophyll layer below, 100×, UV fluorescence.

**Figure 3:**
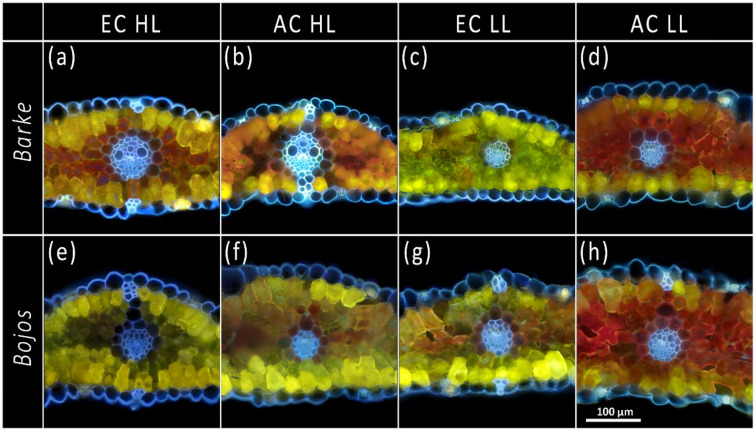
Cross-sections of barley (*Hordeum vulgare)* leaves showing the strong presence of flavonoids in the mesophyll, especially close to leaf surfaces, and the absence of flavonoids in the epidermal pavement cells, 100× magnification, treated with Naturstoff reagent A and acquired under UV light. Epidermal pavement cells exhibit blue fluorescence mainly from outer cell walls, but not from intracellular compartments. Strong yellow fluorescence indicates the presence of flavonoids in mesophyll layers adjacent to both epidermises, with reduced intensity towards the internal leaf tissue. The effect of elevated [CO_2_] on flavonoid accumulation even in the absence of high light is illustrated in (**c**,**g**). Barke (**a**) AC-LL; (**b**) EC-LL; (**c**) AC-HL; (**d**) EC-HL. Bojos (**e**) AC-LL; (**f**) EC-LL; (**g**) AC-HL; (**h**) EC-HL. (Abbreviations are AC-ambient [CO_2_], EC-elevated [CO_2_], HL–high light, LL–low light.).

After treatment with Naturstoff reagent A, three colors of fluorescence were observed when leaves were excited by UV radiation: Red from chlorophyll, blue from autofluorescent PhCs, and yellow from flavonoids reacting with the reagent. Red chlorophyll autofluorescence was detected throughout the mesophyll but was absent in the epidermal pavement cells. Significant blue autofluorescence was observed in vascular bundles, external epidermal cell walls, and sclerenchyma cells associated with the midrib of the leaf (Figure 2d), as well as in stomatal guard cells and epidermal trichomes (Figure 2f). The presence of lignin was confirmed in the vascular bundles via phloroglucinol HCl histochemical detection, however, it was not detected in the epidermis or sclerenchyma cells (Figure 2b). Epidermal pavement cells exhibited blue-fluorescing cell-wall bound PhCs, particularly on the external surface, but lacked any autofluorescence from vacuoles or other organelles. Leaves were examined for presence of the yellow flavonoid fluorescence in the epidermal cells both in cross-section (Figure 2d and Figure 3) and paradermally on leaf segments (Figure 2f,g and Figure S2). The paradermal view on a leaf segment allows epidermal cells to be observed in their intact state while allowing histochemical reagent to penetrate epidermal cells (Figure S2). As with the cross-sections, a faint blue autofluorescence was detected from cell walls of epidermal pavement cells but not from their organelles or central vacuole. The epidermal pavement cells themselves were transparent enough to transmit the yellow flavonoid fluorescence of the mesophyll layers below them (Figure 2d,f,g and Figure S2). The exception was in stomata subsidiary cells, which showed yellow flavonoid fluorescence in vacuoles and cell walls (Figure 2f,g). Subsidiary and guard cells were the only source of flavonoid fluorescence observed from the epidermis. Since epidermal PhCs did not show any changes in localization or intensity of blue autofluorescence across treatments during the pilot screening, only mesophyll PhC accumulation across five leaf depths will be further discussed in results. In the mesophyll cells, PhC fluorescence was detected from cell walls, vacuoles, and chloroplasts (not always simultaneously) (Figure 3).

**Figure 4:**
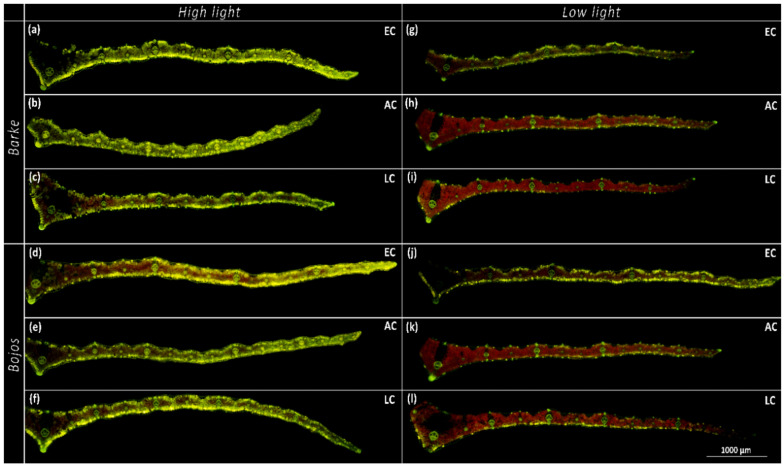
Cross-sections of barley (*Hordeum vulgare*) leaves for each treatment, showing the clear effect of high light intensity, and elevated [CO_2_], on leaf phenolic compounds, 40× magnification, treated with Naturstoff reagent A and excited under blue light–half leaf lamina shown from midrib (left) to leaf margin (right). Left column of images shows high light (HL) treatment, right column shows low light (LL) treatment. [CO_2_] is denoted in frame on right as elevated (EC), ambient (AC), or low (LC). Barke (**a**) EC-LL; (**b**) AC-LL; (**c**) LC-LL; (**g**) EC-HL; (**h**) AC-HL; (**i**) LC-HL. Bojos (**d**) EC-LL; (**e**) AC-LL; (**f**) LC-LL; (**j**) EC-HL; (**k**) AC-HL; (**l**) LC-HL.

**Figure 5:**
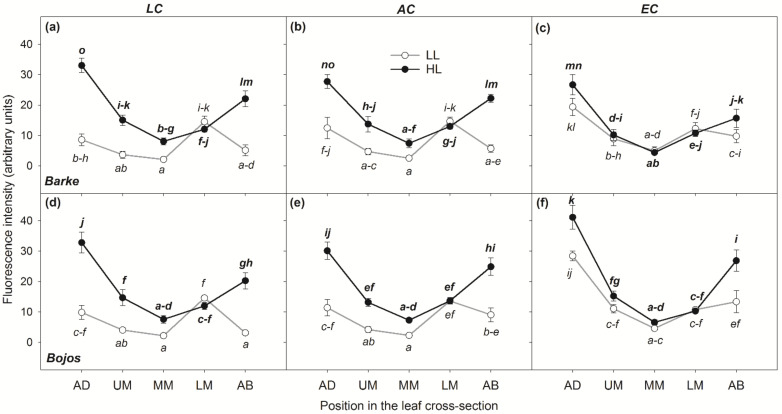
Localization of PhCs in mesophyll tissue of barley (*Hordeum vulgare*) leaf cross section measured by image analysis of fluorescence microscopy. The fluorescence intensity was measured as pixel brightness analyzed in ImageJ software, see Material and Methods. The means (points) and standard errors (error bars) are presented (n = 6). Empty points and grey lines represent low light conditions (LL), black points and black lines represent high light conditions (HL). The graphs in the same line show results for variety Barke (upper, **a**–**c**) and Bojos (lower, **d**–**f**). The graphs in the same column show data for the same growth [CO_2_]: low (LC); ambient (AC); elevated (EC). The following abbreviations are used for localization in mesophyll depth across leaf cross-section: AD–adaxial mesophyll layer, UM–upper mesophyll, MM–middle mesophyll, LM–lower mesophyll and AB–abaxial mesophyll layer. Letters indicate statistically significant differences between means tested by Fishers LSD post hoc test (*p* = 0.05) tested within each variety separately.

As the results of ANOVA show, location within the leaf, together with light intensity, was the most significant factor determining the accumulation of PhCs (*p* < 0.001, Table 1). HL leaves accumulated visibly more PhCs than their LL counterparts (Figure 4). One general pattern was observed for leaves cultivated in either HL or EC conditions: fluorescence intensity from the accumulation of PhCs was generally high in AD mesophyll layer of the leaves, slightly lower in AB mesophyll layer, and a decreasing intensity was observed through both UM and LM with the lowest values found in MM (Figure 5). On the contrary, leaves cultivated in LL and either LC or AC conditions had the highest accumulation of PhCs in the LM rather than the AD mesophyll (although leaves cultivated in LL, but EC followed the same pattern as the HL leaves; Figure 4 and Figure 5).

The effect of light intensity on PhCs accumulation, estimated by image analysis of fluorescence intensity, was highly significant (*p* < 0.001, Table 1) such that plants grown under HL conditions had on average a 46% greater accumulation of PhCs. Specifically, the effect of HL on the accumulation of PhCs was highest and statistically significant in both AD and AB mesophyll layers. Interestingly, for LL cultivation conditions, equal or slightly higher accumulation was found in LM (with the exception of EC leaves) (Figure 5). The differences in PhCs content between LL and HL cultivation conditions decreased in the inner mesophyll layers of the leaves and were statistically insignificant for internal leaf mesophyll positions MM and UM. The differences between HL and LL conditions notably converged with rising [CO_2_]. This interaction between light intensity and [CO_2_] was highly significant, as confirmed by ANOVA (*p* < 0.001, Table 1). Also, highly significant were the interactions between light intensity and [CO_2_] and localization of PhCs within the mesophyll.

The variety showed a significant effect on fluorescence emitted by phenolic compounds on in EC (Figure 5) with Bojos exhibiting higher overall accumulation of PhCs than Barke. On the other hand, the differences between varieties and interactive effects with variety were less significant or insignificant. The only highly significant interaction, including variety, was the interaction with [CO_2_]. This was mainly evident under HL conditions and in AB and AD mesophyll layers. In variety Bojos, EC cultivation increased accumulation of PhCs in AD but not in AB, however, in variety Barke, EC decreased accumulation in AB mesophyll layer (Figure 5).

### 3.2. Target Analysis of Phenolic Compounds by HPLC-HRMS

Specific flavones and phenolic acids were determined by HPLC-HRMS. The phenolic acids are categorized in two groups: hydroxybenzoic acids and hydroxycinnamic acids. The identified compounds were: (1) hydroxybenzoic acids, including 3-hydroxybenzoic acid, protocatechuic acid, vanillic acid, and syringic acid; (2) hydroxycinnamic acids, including 3-coumaric acid, ferulic acid, chlorogenic acid, caffeic acid, and sinapic acid, and (3) flavones, including apigenin, luteolin, isovitexin, homoorientin, and saponarin. Here we discuss mainly the 3 groups, as the microscopic localization of PhCs does not allow discrimination between individual PhCs. However, individual PhC amounts can be found in Table S1.

Among hydroxybenzoic acids, syringic acid in particular was significantly affected by variety, and was increased in both HL and EC leaves. Among hydroxycinnamic acids, a major effect was observed in ferulic, sinapic, and chlorogenic acids. All three were affected by variety (in the opposite way as hydroxybenzoic acids, greater amounts in Bojos), and accumulated more in HL and EC leaves. Among flavones, the dominant effect of treatment was found in saponarin, isovitexin, and homoorientin: higher accumulation under the combination of HL and EC, with almost no differences between varieties.

**Figure 6:**
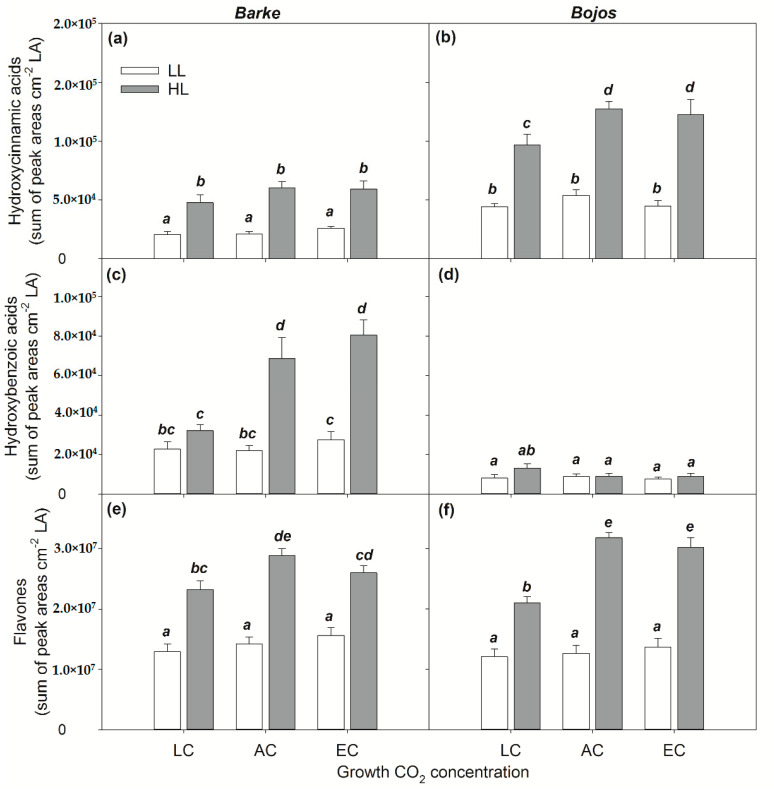
The effect of barley (*Hordeum vulgare)* variety on the accumulation of different groups of PhCs per unit leaf area (LA) under experimental treatment measured by HPLC-HRMS. (**a**,**c**,**e**) relatively tolerant Bojos (**b**,**d**,**f**) oxidative-stress sensitive Barke; Light intensity: white columns-low light (LL); grey columns–high light (HL); and CO_2_ concentration: low (LC), ambient (AC) and elevated (EC) on total amount of (**a**,**b**) hydroxycinnamic acids, (**c**,**d**) hydroxybenzoic acids, and (**e**,**f**) flavones. The means (columns) and standard errors (error bars) are presented (n = 6). Different letters above columns indicate statistically significant differences between means tested by Fisher’s LSD post hoc test (*p* = 0.05) across both varieties.

All three groups of PhCs were significantly affected by the light environment (*p* < 0.001, Table 2). While the accumulation of hydroxycinnamic acids and flavones were significantly increased by HL cultivation irrespective of [CO_2_] treatment and variety, for hydroxybenzoic acids, a significant effect of HL found only in the oxidative-stress sensitive variety Barke in AC or EC conditions (Figure 6). This indicates highly significant (variety × [CO_2_], variety × light, and variety × [CO_2_] × light) or significant ([CO_2_] × light) interactive effects on the content of hydroxybenzoic acids, while for hydroxycinnamic acids the significant interaction was found only in case interaction variety × light. In the case of flavones, significant interactive effects were found for [CO_2_] × light and variety × light (Table 2).

Barley variety showed highly significant effects on hydroxycinnamic acid and hydroxybenzoic acid accumulation, but no significant effect on flavones (Table 2). Hydroxycinnamic acids generally accumulated in higher amounts in the relatively tolerant variety Bojos, while hydroxybenzoic acids were higher in oxidative-stress sensitive variety Barke. Specifically, Barke plants accumulated syringic acid (Table S1), which Bojos plants did not accumulate at all. Flavones accumulated slightly more in variety Bojos, but in comparison of treatment means, the differences between varieties were generally low and mostly insignificant (Figure 6). Significant differences in flavones between varieties were found only under EC and HL conditions.

The effect of [CO_2_] on accumulation of all three groups of PhCs, (Table 2; Figure 6) was statistically significant (*p* < 0.05) only under HL conditions. The major differences were found between LC/AC and EC, however, the responses to [CO_2_] were variety specific. While higher effect of [CO_2_] on hydroxycinnamic acids and flavones was found in variety Bojos, variety Barke showed an increase of hydroxybenzoic acids in response to [CO_2_] (Figure 6). In general, the differences between AC and EC in accumulation of all three groups of PhCs were small and statistically insignificant. The interactive effects of light intensity and [CO_2_] show that both LL and LC plants accumulated less PhCs, irrespective of barley variety (Figure 6).

### 3.3. Chlorophyll and UV Screening Indices

Both chlorophyll index and UV screening index were significantly affected by barley variety, [CO_2_], and light intensity (Table 2). However, statistically significant interactions were found only for the UV screening index. These were highly significant (*p* < 0.01) for interactions variety × light, [CO_2_] × light and variety × [CO_2_] (Table 2).

**Figure 7:**
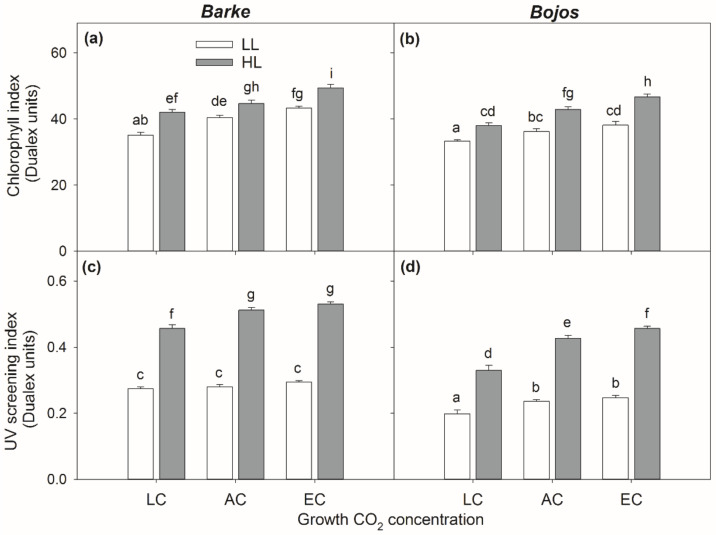
The effect of barley (*Hordeum vulgare)* variety on chlorophyll index and UV screening index under different experimental treatments. (**a**,**c**) Barke, (**b**,**d**) Bojos, light intensity (white columns-low light (LL); grey columns–high light, (HL) and CO_2_ concentration [CO_2_]: low (LC), ambient (AC), elevated (EC) on chlorophyll index (**a**,**b**) and UV screening index (**c**,**d**) determined in vivo using instrument Dualex. The means (columns) and standard errors (error bars) are presented (n = 6). Different letters above columns indicate statistically significant differences between means tested by Fishers LSD post hoc test (*p* = 0.05) across both varieties.

HL significantly increased both chlorophyll index and UV screening index across all treatments independently on variety (Figure 7). The effect of light on the UV screening index slightly increased with increasing [CO_2_]. The oxidative-stress sensitive variety Barke showed generally higher chlorophyll index and particularly UV screening index comparing to relatively tolerant Bojos. The differences between varieties were significant for UV screening index in all [CO_2_] and light treatments, while the differences in chlorophyll index between varieties were less evident and only statistically significant under EC (both HL and LL), AC (only LL), LC (only HL). Increasing [CO_2_] generally increased both the chlorophyll index and UV screening index. While this effect was evident for the chlorophyll index under both light intensities, the effect of [CO_2_] was more pronounced for the UV screening index under HL.

### 3.4. Relationships between Localization and Accumulation of PhCs in Leaves

**Figure 8:**
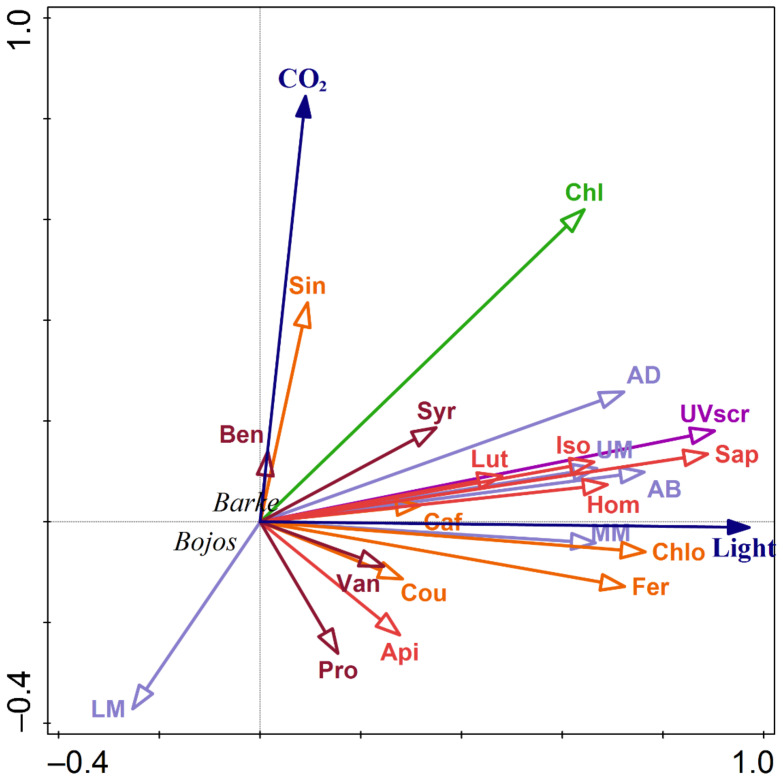
Biplot diagram representing results of redundancy analysis (RDA) on the effects of light intensity (Light), CO_2_ concentration (CO_2_), barley variety (Barke, Bojos) on the localization of PhCs within leaf cross-section (light purple; AD—adaxial mesophyll layer, UM—upper mesophyll, MM—middle mesophyll, LM—lower mesophyll and AB—abaxial mesophyll layer), content of hydroxycinnamic acids (orange; Cou—3-coumaric acid, Caf—caffeic acid, Fer—ferulic acid, Sin—sinapic acid, Chlo—chlorogenic acid), hydroxybenzoic acids (brown; Ben—3-hydroxybenzoic acid, Pro—protocatechuic acid, Van—vanillic acid, Syr—syringic acid), flavones (red; Api—apigenin, Lut—luteolin, Iso—isovitexin, Hom—homoorientin, Sap—saponarin), chlorophyll index (Chl) and UV screening of chlorophyll fluorescence (UVscr). Explained variation by axis 1 = 82.8% and cumulative explained variation by both axis = 91.6% (Pseudo-F = 25.9, *p* = 0.002).

The associations between environmental drivers ([CO_2_], light intensity) and accumulation of PhCs, either expressed as total amounts of individual groups of PhCs expressed per unit leaf area or their accumulation within leaf cross-sections assessed by Naturstoff reagent A fluorescence, were tested using redundancy analysis (RDA, Figure 8). Most parameters related to the accumulation of PhCs were affected primarily by light intensity during cultivation. As an exception, the effect of light was lower for hydroxybenzoic acid accumulation and reversed for PhC accumulation in the LM compared to other locations in leaf cross-section or total content of other groups of PhCs. Accumulation in the lower mesophyll was also inversely related to [CO_2_]. Increasing [CO_2_] also positively affected the chlorophyll index (alongside the positive effect of light intensity).

**Figure 9:**
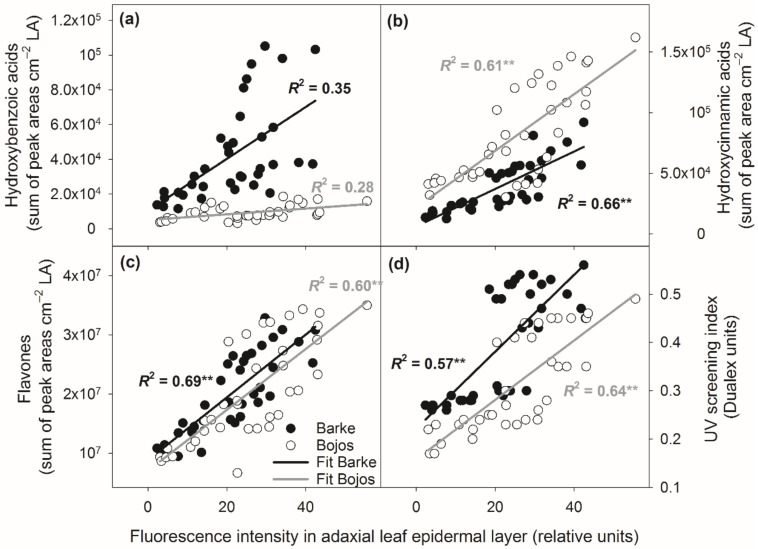
Linear relationships between accumulation of different groups of PhCs in adaxial leaf epidermal layer measured as fluorescence intensity under blue light excitation and total amount of (**a**) hydroxybenzoic acids, (**b**) hydroxycinnamic acids, (**c**) flavones, and (**d**) UV screening index. Amounts of hydroxybenzoic acids, hydroxycinnamic acids, and flavones are expressed per unit leaf area (LA). Relationships are separated by barley (*Hordeum vulgare)* variety: Barke–black points and black lines, Bojos–empty points and grey lines. The index of determination (*R*^2^) and significance of Pearson’s correlation coefficient (** significant at *p* = 0.01).

Close relationships between PhC localization in adaxial leaf surface and accumulation of individual groups of PhCs confirmed the results of RDA, however, variety had an important effect (Figure 9). For the same fluorescence intensity in AD, variety Barke accumulated more hydroxybenzoic acids and flavones, and less hydroxycinnamic acids. Barke also showed a higher UV screening index for the same fluorescence intensity in AD compared to variety Bojos. The varieties show fundamentally different relationships for hydroxybenzoic acids. While almost no relationship was found for Bojos, in Barke the relationship between fluorescence intensity in AD mesophyll and hydroxybenzoic acids was much steeper, although not statistically significant.

## 4. Discussion

PhCs are known to play a role in many biotic and abiotic stress responses, particularly by functioning as antioxidants to scavenge ROS and screen UV radiation [18,23,26,28]. Visualizing changes in PhC localization and accumulation is the next step for understanding how leaves respond to environmental conditions. This paper presents an original method for measuring the relative accumulation of PhCs throughout leaf tissue layers (Figure 1). We are aware that due to heterogeneity in excitation wavelength penetration into the leaf tissues [45] (also known as a sieve effect [42,46,47]) our proposed approach cannot replace quantification of PhCs, such as fluorescence spectroscopy or HPLC-HRMS, which were also used in the present study. However, the presented method can add another dimension for analysis of PhCs in leaf tissues, while localization may contribute to the understanding of their functional role in the plant defense mechanisms.

PhC detection in leaf cross-sections brought us to the surprising conclusion that PhC accumulation in response to environmental inputs, represented by [CO_2_] and light intensities, occurred primarily in the mesophyll of barley instead of the epidermis (Figure 2d,f,g, Figure 3 and Figure S2). This finding is contrary to the idea that PhCs localize primarily in the vacuoles of epidermal cells for UV screening, as has been commonly shown for other species, such as *Ginkgo biloba* [48] and *Kalanchoë daigremontiana* [49]. By contrast, our results show only cell-wall bound PhCs along the outer epidermis and in thorn-like extensions, and no visible signal from epidermal pavement cell vacuoles (Figure 2d,f,g and Figure 3).

PhCs are primarily synthesized via a multi-enzyme complex localized in the cytoplasmatic surface of the endoplasmic reticulum and then transported either to the vacuole or to the cell wall [50]. Cell wall PhCs play a role in tolerance to pathogens [51], and screening UV radiation [12,52], while vacuolar PhCs provide a reservoir for antioxidant functions—although physically separated from the source of ROS production in chloroplasts [21]. In the chloroplast, O_2_^−^ is quickly converted to H_2_O_2_, a diffusible oxidant which can then diffuse into the vacuole and be scavenged by PhCs [53]. The transport of PhCs to the vacuole is a key prerequisite for their ongoing biosynthesis as it creates a sink for assimilated carbon [54]. Differing fluorescence patterns under UV excitation indicate that different groups of PhCs accumulate in the epidermal pavement cell walls (blue fluorescence characteristic of cell-wall bound hydroxycinnamic acid derivatives) compared to the mesophyll cell walls, vacuoles, and chloroplasts (yellow fluorescence characteristic of flavonoids) (Figure 2d and Figure 3). This is supported by the claim that hydroxycinnamic acid derivatives and flavonoids may have different roles in responding to high-intensity light, i.e., that HCA derivatives are better at UV screening (and localize close to the leaf surface-epidermis) but that flavonoids serve as free radical scavengers induced by excess light (and therefore localize around cells with active photosynthesis, i.e., in the mesophyll) [55].

According to Hutzler et al. [25], UV screening is mainly achieved by hydroxycinnamic acids and flavonoids in the epidermis, primarily in the cuticle, cell walls, or vacuole. However, studies in herbaceous plants show that UV-B still penetrated the anticlinal cell walls of the epidermis and reached the mesophyll [45], implying the need for protective PhCs even below the epidermis. A study by Liu et al. [56] showed that at least 50% of barley flavonoids occurred in the lower mesophyll rather than in the epidermis. This study used HPLC analysis on epidermal peels and isolated mesophyll tissue–although the presence of flavonoids in barley mesophyll was confirmed, the gradient from outer mesophyll (epidermis-adjacent) to middle mesophyll was not described. Studies in rye (*Secale cereale*) showed both epidermal hydroxycinnamic acids and mesophyll flavonoids were constitutive, while epidermal flavonoids increased with age and light intensity [57]. This is contrary to our finding that hydroxycinnamic acids were increased by high light intensity (Figure 6) and that flavonoids were mostly absent from the epidermis (except in stomata) while flavonoids in the mesophyll had a strong light-responsive and [CO_2_]- responsive presence (Figure 2d and Figure 3). This discrepancy could indicate a greater variety in how PhC-mediated stress response occurs in leaves of different species. For instance, dicotyledonous soybeans (*Glycine max*) are known to accumulate flavonoids only in the epidermis, while monocotyledonous oats (*Avena sativa*) accumulate flavonoids also in the mesophyll [56,58]. Another study, also using Naturstoff reagent A, found varying PhC localization even between closely related species of Vaccinium: notably, one species allocated the majority of PhCs to the outer surface of the epidermis, while another species only had PhCs associated with stomatal guard cells, but a greater localization in the palisade mesophyll layer [59]. This study further noted that while total phenolic compositions varied, the blue fluorescent signal from cell-wall bound PhCs in the epidermis remained constant (also observed by Lichtenthaler and Schweiger [40]). The most prevalent cell-wall bound PhCs are hydroxycinnamic acids [40,60] and specifically ferulic acid in Poaceae [61]. The observed primary presence of PhCs in the mesophyll, rather than in the epidermis, of barley leaves is a relatively unique result which suggests that leaves of different species and phenological stages present varied PhC-modulated leaf protective responses. In fact, later in the ontogeny of Bojos variety plants, occasional presence of flavonoids in some epidermal pavement cells was observed after exposure to stressors, such as drought and high temperature (results not shown). Clearly, flavonoids can occur in barley epidermal pavement cells, although they were not detected under the conditions of the present experiment.

Our results showed that light intensity is the major factor in terms of accumulation and localization of total PhCs (Figure 4, Table 1), with higher light intensity resulting in higher accumulation of PhCs in the mesophyll, particularly in cells adjacent to the epidermis (Figure 3 and Figure 5). Leaves grown in low light showed accumulation of PhCs deeper in the mesophyll at low and ambient CO_2_ (Figure 5), but fewer PhCs overall (Figure 4 and Figure 6). EC reduced the differences between HL and LL treatments and promoted an accumulation pattern similar to HL (Figure 3, Figure 4 and Figure 5). The flavones (luteolin, isovitexin, homoorientin and saponarin) were strongly associated with UV screening index (Figure 8). The UV screening index indicates that the accumulation and localization, especially of ortho-dihydroxylated flavonoids, increases with increasing [CO_2_] at HL (Figure 7), a result noted in other plants, such as *Betula pendula* [62]. Strong antioxidant flavones, luteolin, and homoorientin, were accumulated much more in HL leaves, but not to the same degree in EC leaves (Table S1). Similarly, HL induced the increase of selected flavonoids in other barley varieties [63]. Our results show that high light increased both flavones and hydroxycinnamic acids similarly (Figure 6), although certain compounds with poor antioxidant qualities (coumaric acid, apigenin) showed a weaker association with light intensity than other compounds (Figure 8). As reported by Kowalczewski et al. [63], the high-light induced increase of hydroxycinnamic acids in barley was slightly more pronounced than the increase for flavones. Several studies report that high light + UV increases the ratio of flavonoids to hydroxycinnamic acids, which is interesting as hydroxycinnamic acids and their derivatives are more effective in UV-B screening, while flavonoids are more effective antioxidants [23,55,64]. It has been proposed that the accumulation of flavonoids may effectively protect sensitive tissues from photooxidative damage via intercellular scavenging of ROS [65]. Another highly attractive, and not mutually exclusive hypothesis, is that the biosynthesis of flavonoids serves as an alternative use for excess photosynthetic energy under conditions of high light or high C:N ratios. PhC biosynthesis may sustain photosynthesis under stress by consuming reducing power and excess carbon intermediates [22]. In this case, PhCs could theoretically be evenly distributed throughout the mesophyll, however, EC leaves still accumulated the greatest amount of PhCs near to leaf surfaces.

This study investigated barley leaves cultivated in various levels of atmospheric [CO_2_]. Plant responses to EC are interesting in terms of how future plants will fare, while LC cultivation addresses mechanisms of carbon limitation, as well as questions of past ecophysiological function—200 ppm is representative of the low levels of atmospheric [CO_2_] experienced by land plants during the last glacial period [66,67]. Extending the range of [CO_2_] allows for a better understanding of its role in plant secondary metabolism. The effect of carbon limitation may not be seen at AC or EC, especially if other factors such as low light intensity or low nitrogen availability are reducing photosynthetic capacity [68,69]. The role of [CO_2_] on secondary metabolism is not fully clarified yet and shows compound-specific responses and dependence on other environmental conditions such as light intensity [70]. Moreover, the plant response to [CO_2_] regarding carbon allocation to PhCs may be different for annual plants such as barley versus woody species. A study on Norway spruce [71] showed that trees grown under elevated [CO_2_] invested the extra carbon rather to stem and shoot growth than to accumulation of PhCs in needles. Our findings show that barley plants grown in EC benefit from the excess available [CO_2_] to synthesize PhCs to a higher degree than plants grown in equivalent light conditions in AC and particularly in LC (Figure 3 and Figure 5). This effect is more evident under LL conditions, so it seems that elevated [CO_2_] is partly able to replace the role of HL in the accumulation of PhCs. The result is a convergence of differences between LL and HL conditions with increasing [CO_2_]. The positive effect of elevated [CO_2_] on PhC metabolism was already proven in recent studies by Ibrahim et al. and Peñuelas et al. [29,72].

Although the mechanisms remain unclear, Ibrahim et al. [29] showed tight relationships between photosynthetic rate and accumulation of PhCs. Carbon-rich PhCs form a substantial carbon sink for photosynthetic assimilates and may reduce the occurrence of feedback down-regulation of photosynthesis under elevated [CO_2_] [72]. What is curious about the localization data for EC barley leaves grown in LL versus HL is that both groups accumulate PhCs near to the surface, rather than in the lower mesophyll as seen for other LL treatments. Such a pattern of PhC localization can be attributed to increasing chlorophyll content per area unit due to increasing [CO_2_] and high light (Figure 7) and by an increase in leaf thickness mainly due to high light. Both factors can reduce the light transmittance to the middle mesophyll and thus results in higher gradients within leaf cross-section. Positive effect of both light intensity and [CO_2_] on leaf thickness was documented by meta-analysis across several species [73]. an increasing chlorophyll content under both high [CO**_2_**] and light intensity was also documented in wheat by Yi et al. [74].

Overall, the PhC content was significantly influenced by barley variety, especially for hydroxycinnamic acids and hydroxybenzoic acids (Table 2). Previous studies already demonstrated lower constitutive PhCs (under UV exclusion and low light conditions) in Barke variety compared to another tolerant genotype (Bonus) [75]. However, high light led to an accumulation of PhCs exceeding the constitutive contents several fold. In our study, the Bojos variety showed a higher accumulation of hydroxycinnamic acids, while the Barke variety had a higher accumulation of hydroxybenzoic acids (Figure 6), particularly syringic acid, which was absent in the Bojos variety (Table S1). In other studies, PhCs profile was shown to be variety dependent and among environmental factors, timing of water scarcity had stronger effect on PhCs than light intensity [63]. Syringic acid levels, in particular, can be a distinguishing trait among barley varieties [76]. Between the two groups of phenolic acids, the hydroxycinnamic acids are usually cited as being stronger antioxidants than hydroxybenzoic acids [20]. While hydroxycinnamic acids play a role in UV screening, hydroxybenzoic acids may promote resistance to biotic stressor. Barley leaves treated with vanillic, isovanillic, or syringic acid showed a reduction in mildew by more than 80% [77]. The higher accumulation of hydroxybenzoic acids in the Barke variety may explain its purported tolerance to common biotic stressors [78], including leaf rust, leaf scald, net blotch, and mildew, while maintaining its status as oxidative-stress sensitive. Barke also showed higher accumulation of ortho-dihydroxylated flavonoids, as indicated by the UV screening index (Figure 7) which is associated mainly to the accumulation of ortho-dihydroxylated flavonoids [42]. The accumulation of antioxidant PhCs in stress-sensitive plants may be the result of less efficient initial defenses to ROS production, and thus exposure to more severe states of oxidative stress [23]. Meanwhile, the high level of hydroxycinnamic acids in leaves of Bojos plants provide better protection from high light and a possible lower stress load on the plant. Additionally, the Bojos variety accumulated more PhCs in epidermal-adjacent mesophyll cell layers, especially under EC conditions (Figure 5). These data could lead to a hypothesis that certain groups of PhCs (i.e., hydroxycinnamic acids) and certain mesophyll localizations (i.e., adjacent to epidermal leaf surfaces) provide increased protection from direct photooxidative stress as a product of high light.

In conclusion, our findings show that high light intensity enhanced the accumulation of nearly all PhCs detected by HPLC-HRMS and promoted their localization in the epidermal-adjacent mesophyll layers (Figure 4 and Figure 5). Elevated [CO_2_] also increased PhC accumulation, even in the absence of high intensity light (Figure 3 and Figure 5). Additionally, barley varieties with varying tolerance to oxidative stress showed differences in their respective levels of hydroxycinnamic acids and hydroxybenzoic acids. Oxidative-stress sensitive variety Barke accumulated more flavones and hydroxybenzoic acids, particularly syringic acid, which was absent in Bojos (Table S1), while the variety Bojos accumulated more hydroxycinnamic acids (Figure 6). We hypothesize that the greater antioxidative efficiency provided by the CH=CH-COOH group in hydroxycinnamic acids compared to the COOH group in hydroxybenzoic acids may be responsible for the higher tolerance of oxidative stress in the Bojos variety.

In addition, we believe that our study brings an original multidisciplinary aspect to the study of PhC accumulation and localization. The combination of histochemical detection on leaf cross sections with image analysis in tissue layers allows for a more complex, more detailed evaluation of the localization of detected compound in gradients across organs and tissues. This approach could be valuable not only in plant histochemistry but in animal and human studies as well.

## Figures and Tables

**Table 1:** Results of four-way ANOVA on the effects of barley variety (Var), CO_2_ concentration ([CO_2_]), light intensity (Light), and tissue localization (Loc) in leaf cross-sections, and their mutual interactions (×) on fluorescence intensity emitted by phenolic compounds enhanced with Naturstoff reagent A showing localization of within leaf cross-sections. Degrees of freedom (*df*), *F* and *p* values shown for each factor and interaction. Significant effects (*p* < 0.05) are indicated in bold.

	*df*	*F*	*p*
Var	1	10.09	**0.002**
[CO_2_]	2	7.85	**<0.001**
Light	1	266.23	**<0.001**
Loc	4	147.23	**<0.001**
Var × [CO_2_]	2	8.82	**<0.001**
Var × Light	1	2.01	0.157
[CO_2_] × Light	2	15.15	**<0.001**
Var × Loc	4	3.00	**0.019**
[CO_2_] × Loc	8	5.92	**<0.001**
Light × Loc	4	44.58	**<0.001**
Var × [CO_2_] × Light	2	1.67	0.189
Var × [CO_2_] × Loc	8	2.33	**0.019**
Var × Light × Loc	4	0.17	0.955
[CO_2_] × Light × Loc	8	2.12	**0.034**
Var × [CO_2_] × Light × Loc	8	0.24	0.982

**Table 2:** Results of three-way ANOVA on the effects of barley (*Hordeum vulgare)* variety (Var), CO_2_ concentration ([CO_2_]), light intensity (Light), and their mutual interactions on total hydroxybenzoic acids, hydroxycinnamic acids, and flavones determined by HPLC-HRMS and on chlorophyll and UV screening index measured in vivo using the instrument Dualex. Degrees of freedom (*df*), *F* and *p* values are denoted for each factor or interaction. Significant effects (*p* < 0.05) are indicated in bold.

		Hydroxybenzoic Acids		Hydroxycinnamic Acids		Flavones		Chlorophyll Index		UV Screening Index	
	***df***	***F***	***p***	***F***	***p***	***F***	***p***	***F***	***p***	***F***	***p***
Var	1	167.03	**<0.001**	140.09	**<0.001**	0.02	0.895	44.94	**<0.001**	223.92	**<0.001**
[CO_2_]	2	7.73	**0.001**	5.21	**0.008**	15.28	**<0.001**	73.39	**<0.001**	62.87	**<0.001**
Light	1	56.98	**<0.001**	198.83	**<0.001**	325.94	**<0.001**	160.55	**<0.001**	1517.14	**<0.001**
Var × [CO_2_]	2	11.15	**<0.001**	1.21	0.305	1.22	0.302	0.43	0.653	6.72	**0.002**
Var × Light	1	44.61	**<0.001**	23.16	**<0.001**	4.43	**0.040**	0.74	0.394	14.83	**<0.001**
[CO_2_]× Light	2	5.72	**0.005**	2.22	0.117	8.05	**<0.001**	1.49	0.234	16.13	**<0.001**
Var × [CO_2_] × Light	2	8.67	**<0.001**	0.58	0.562	2.35	0.104	2.40	0.099	0.53	0.592

## Data Availability

Data available upon request.

## Supplementary Materials

The following are available online at https://www.mdpi.com/2076-3921/10/3/385/s1 Figure S1: Diurnal courses of air temperature, relative air humidity, photosynthetically active and UV-A radiation inside the growth chambers during the experiment. Figure S2: Paradermal images of barley (*Hordeum vulgare*) leaf segments demonstrating the absence of PhCs detected by Naturstoff reagent A in the epidermal pavement cells. Table S1: Levels of specific PhCs levels measured using HPLC-HRMS.
