# Supplementary material for: Light and CO2 Modulate the Accumulation and Localization of Phenolic Compounds in Barley Leaves
Source: Antioxidants (Basel). 2021 Mar 5;10(3):385. doi: 10.3390/antiox10030385 (PMC7999350; doi:10.3390/antiox10030385)
Supplement: Supplementary file 1 [file antioxidants-10-00385-s001.zip › Supplementary/Supplementary material.docx]

**Supplementary Materials:** The following are available online at www.mdpi.com/xxx/s1. Figure S1: Diurnal courses of air temperature, relative air humidity, photosynthetically active and UV-A radiation inside the [growth chambers](https://www.sciencedirect.com/topics/agricultural-and-biological-sciences/growth-chambers) during the experiment. Figure S2: Paradermal images of barley (*Hordeum vulgare*) leaf segments demonstrating the absence of PhCs detected by Naturstoff reagent A in the epidermal pavement cells. Table S1: Level of specific PhCs levels measured using HPLC-HRMS

**
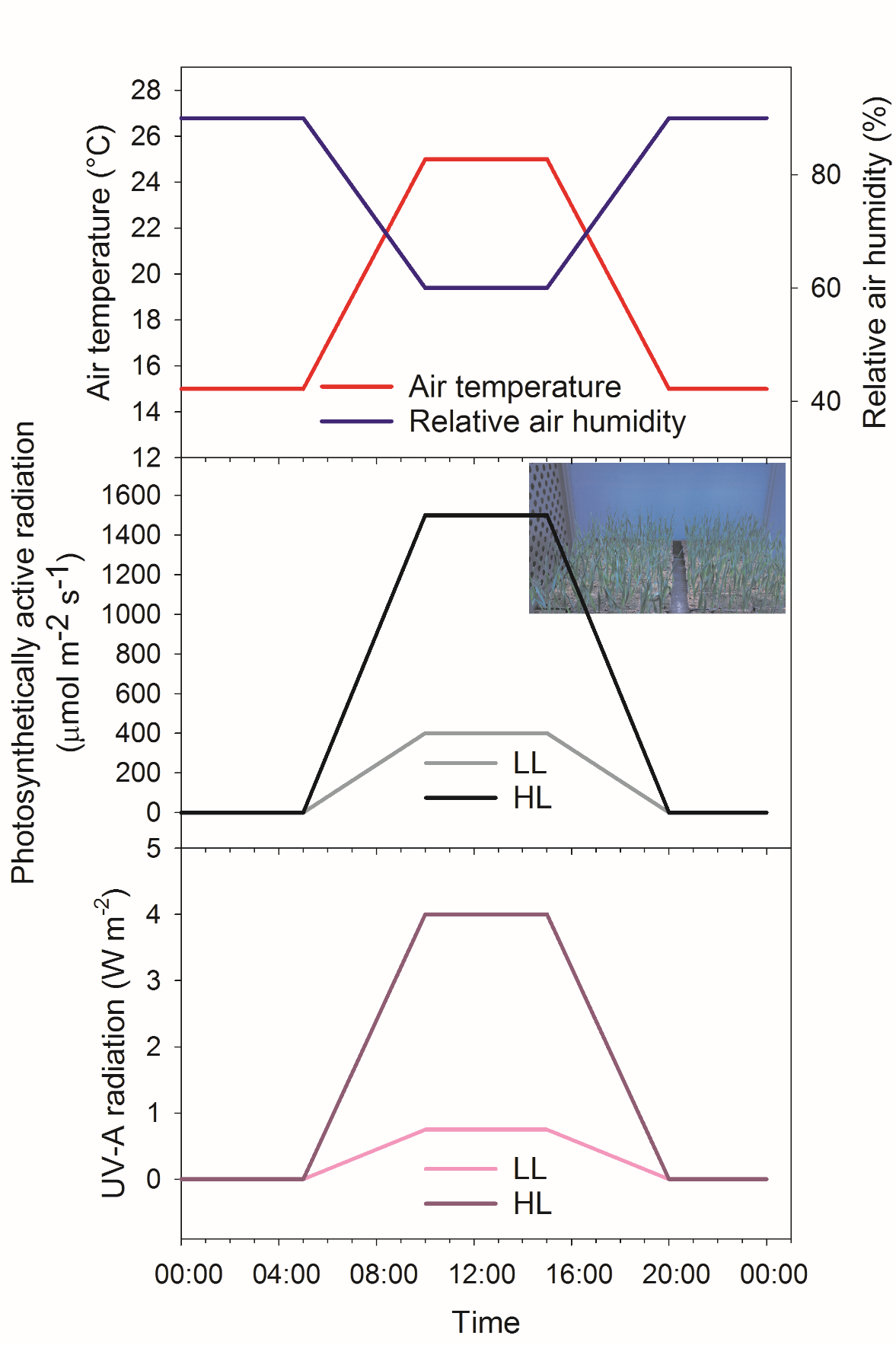
Figure S1**: Diurnal courses of air temperature, relative air humidity, photosynthetically active and UV-A radiation inside the [growth chambers](https://www.sciencedirect.com/topics/agricultural-and-biological-sciences/growth-chambers) during the experiment. A view on experimental plants inside a growth chamber is presented in the middle compartment.

**
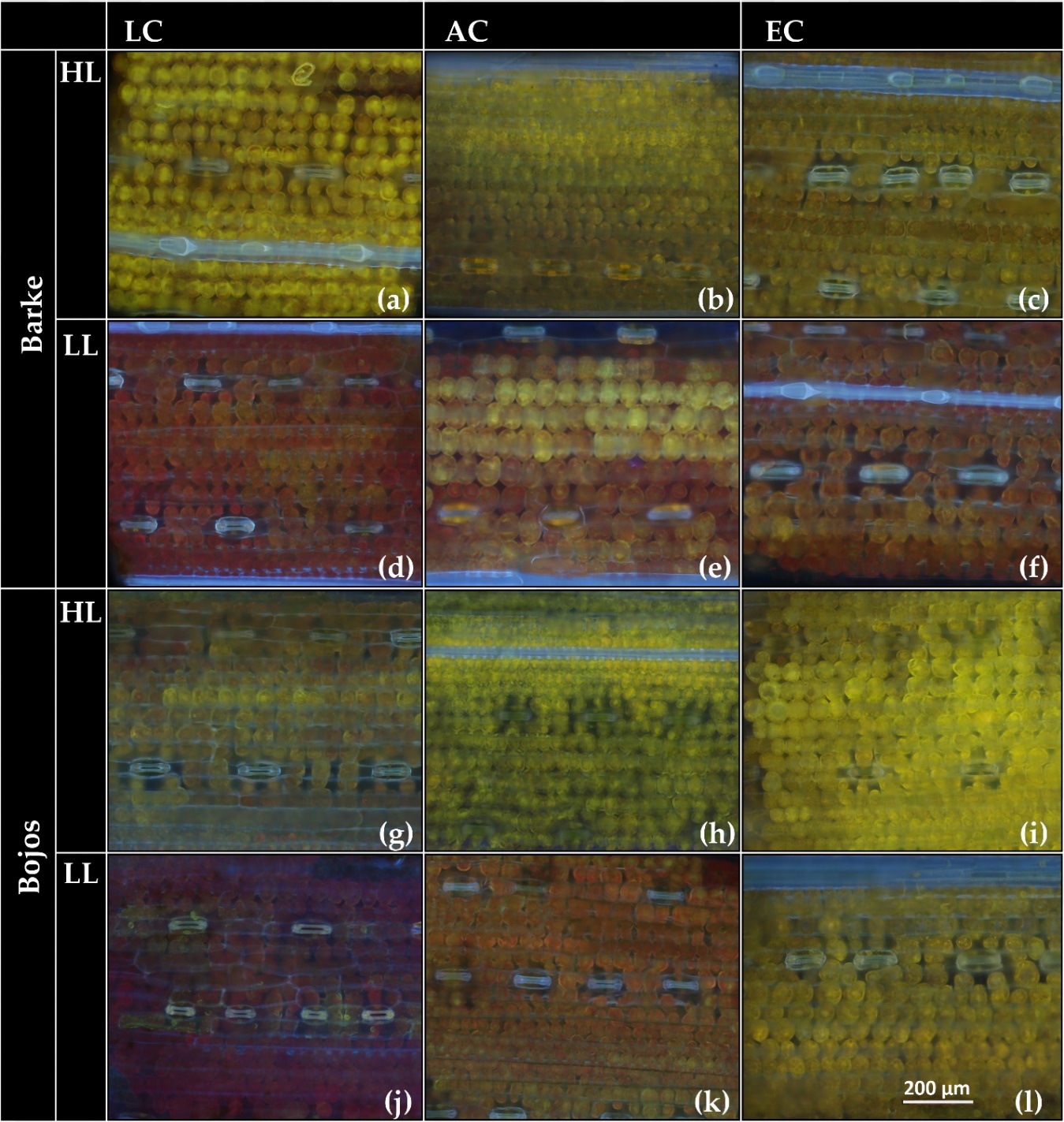
Figure S2**: Paradermal images of barley (*Hordeum vulgare*) leaf segments demonstrating the absence of PhCs (flavonoids) detected by Naturstoff reagent A in the epidermal pavement cells, and their abundant presence in the mesophyll layer adjacent to the epidermis. Flavonoids in the epidermis are present only in stomatal subsidiary and guard cells. Magnification 10x, UV fluorescence: Red from chlorophyll autofluorescence, blue from autofluorescent PhCs, and yellow from flavonoids reacting with the Naturstoff reagent A. Barke variety: a.) HL-LC, b.) HL-AC, c.) HL-EC, d.) LL-LC, e.) LL-AC, f.) LL-EC. Bojos variety: g.) HL-LC, h.) HL-AC, i.) HL-EC, j.) LL-LC, k.) LL-AC, l.) LL-EC. Low light (LL) and high light (HL); CO_2_ concentration: low (LC), ambient (AC) and elevated (EC).

**Supplementary Table S1**: Means of individual PhCs amounts (peak areas cm^-2^) as measured HPLC-HRMS analysis. Homogeneous groups (letters) analysed by Fischers LSD post-hoc test (p = 0.05) following a three-way ANOVA across both barley varieties: Barke (relatively tolerant to oxidative stress) and Bojos (sensitive to oxidative stress). Light intensity: low light (LL) and high light (HL). CO_2_ concentration: low (LC), ambient (AC) and elevated (EC).

|  | **Barke** | | | | | | | | | | | |
| --- | --- | --- | --- | --- | --- | --- | --- | --- | --- | --- | --- | --- |
|  | **LL** | | | | | | **HL** | | | | | |
|  | **LC** | | **AC** | | **EC** | | **LC** | | **AC** | | **EC** | |
| **Hydroxybenzoic acids** | | |  |  |  |  |  |  |  |  |  |  |
| 3-hydroxybenzoic acid | 605.3 | ab | 598.2 | ab | 799.0 | ab | 746.4 | ab | 316.9 | ab | 434.3 | ab |
| Protocatechuic acid | 388.8 | b | 179.9 | ab | 0 | a | 295.0 | ab | 386.0 | b | 209.0 | ab |
| Vanillic acid | 3205.9 | ab | 3726.0 | ab | 4736.6 | abc | 3823.5 | ab | 5061.3 | abc | 5555.9 | abc |
| Syringic acid | 18583.3 | b | 17480.1 | b | 21967.7 | b | 27248.8 | b | 62794.4 | c | 74171.5 | c |
|  |  |  |  |  |  |  |  |  |  |  |  |  |
| **Hydroxycinnamic acids** | | |  |  |  |  |  |  |  |  |  |  |
| 3-coumaric acid | 0 | a | 68.7 | ab | 20.8 | a | 53.7 | ab | 96.3 | ab | 31.2 | ab |
| Caffeic acid | 97.6 | ab | 0 | a | 0 | a | 65.3 | a | 236.4 | abc | 173.7 | ab |
| Ferulic acid | 5322.5 | a | 6005.4 | a | 7101.7 | ab | 14022.3 | bc | 17572.5 | cd | 22577.4 | d |
| Sinapic acid | 12622.0 | ab | 11260.6 | ab | 17344.8 | bc | 9150.1 | a | 14109.9 | ab | 23439.3 | c |
| Chlorogenic acid | 2442.8 | a | 3598.7 | ab | 1703.6 | a | 24410.5 | c | 28360.5 | c | 13232.3 | b |
|  |  |  |  |  |  |  |  |  |  |  |  |  |
| **Flavones** |  |  |  |  |  |  |  |  |  |  |  |  |
| Apigenin | 3328.8 | abc | 3903.7 | abc | 3261.5 | abc | 7594.2 | cd | 3756.9 | abc | 2143.6 | ab |
| Luteolin | 0 | a | 0 | a | 0 | a | 323.0 | a | 983.6 | b | 198.3 | a |
| Isovitexin | 16353.3 | a | 19365.0 | ab | 19961.6 | ab | 41350.9 | c | 61585.6 | d | 37536.0 | bc |
| Homoorientin | 592.3 | a | 2569.1 | a | 1263.4 | a | 40369.2 | bc | 170978.9 | e | 57630.0 | c |
| Saponarin | 12892920 | ab | 14162042 | ab | 15603123 | b | 23135463 | cd | 28607367 | ef | 25916772 | de |
|  | **Bojos** | | | | | | | | | | |  |
|  | **LL** | | | | | | **HL** | | | | |  |
|  | **LC** | | **AC** | | **EC** | | **LC** | | **AC** | | **EC** | |
|  |  |  |  |  |  |  |  |  |  |  |  |  |
| **Hydroxybenzoic acids** | | |  |  |  |  |  |  |  |  |  |  |
| 3-hydroxybenzoic acid | 703.7 | ab | 71.0 | a | 943.9 | ab | 235.0 | a | 1161.7 | b | 950.2 | ab |
| Protocatechuic acid | 210.1 | ab | 274.0 | ab | 128.9 | ab | 169.3 | ab | 366.1 | b | 239.7 | ab |
| Vanillic acid | 6386.9 | abc | 7437.4 | c | 5371.4 | abc | 11750.3 | d | 6830.0 | bc | 7594.4 | c |
| Syringic acid | 772.9 | a | 1093.2 | a | 1137.1 | a | 1067.3 | a | 457.1 | a | 130 | a |
|  |  |  |  |  |  |  |  |  |  |  |  |  |
| **Hydroxycinnamic acids** | | |  |  |  |  |  |  |  |  |  |  |
| 3-coumaric acid | 49.7 | ab | 40.7 | ab | 78.5 | ab | 281.6 | c | 60.7 | ab | 156.8 | bc |
| Caffeic acid | 135.7 | ab | 485.1 | abcd | 140.4 | ab | 619.2 | bcd | 755.6 | cd | 950.4 | d |
| Ferulic acid | 14500.2 | bc | 10792.0 | abc | 5926.9 | a | 38254.1 | e | 36795.3 | e | 31219.8 | e |
| Sinapic acid | 23517.7 | c | 31565.5 | d | 33358.2 | d | 23704.8 | c | 34454.6 | d | 36291.4 | d |
| Chlorogenic acid | 6058.0 | ab | 11061.9 | ab | 5426.6 | ab | 34047 | c | 55205.0 | d | 54067.4 | d |
|  |  |  |  |  |  |  |  |  |  |  |  |  |
| **Flavones** |  |  |  |  |  |  |  |  |  |  |  |  |
| Apigenin | 4622.8 | abcd | 3996.0 | abc | 895.2 | a | 5847.7 | bcd | 5234.6 | abcd | 8454.2 | d |
| Luteolin | 0 | a | 0 | a | 0 | a | 0 | a | 1062.6 | b | 1064.9 | b |
| Isovitexin | 19502.8 | ab | 20732.2 | ab | 14211.0 | a | 26472.6 | abc | 71487.5 | d | 80958.2 | d |
| Homoorientin | 352.8 | a | 2112.4 | a | 888.3 | a | 12404.5 | ab | 121338 | d | 95136.8 | d |
| Saponarin | 12032798 | a | 10945739 | a | 13645148 | ab | 20998700 | c | 31603989 | f | 30008883 | f |
