# Supplementary figures and images for: Light and CO2 Modulate the Accumulation and Localization of Phenolic Compounds in Barley Leaves
Source: Antioxidants (Basel). 2021 Mar 5;10(3):385. doi: 10.3390/antiox10030385 (PMC7999350; doi:10.3390/antiox10030385)

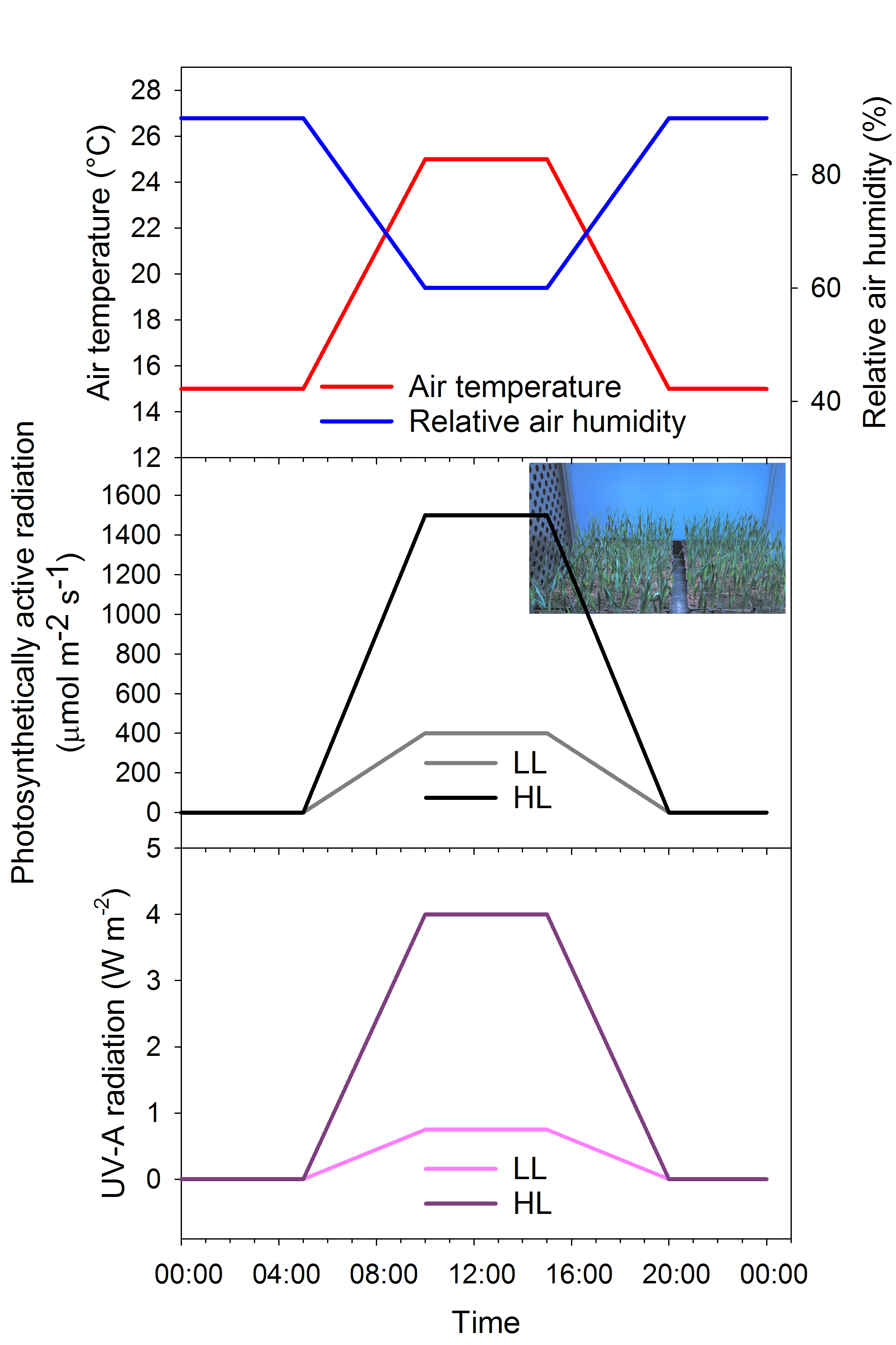

Supplement: Supplementary file 1 [file antioxidants-10-00385-s001.zip › Supplementary/Fig S1.TIF]

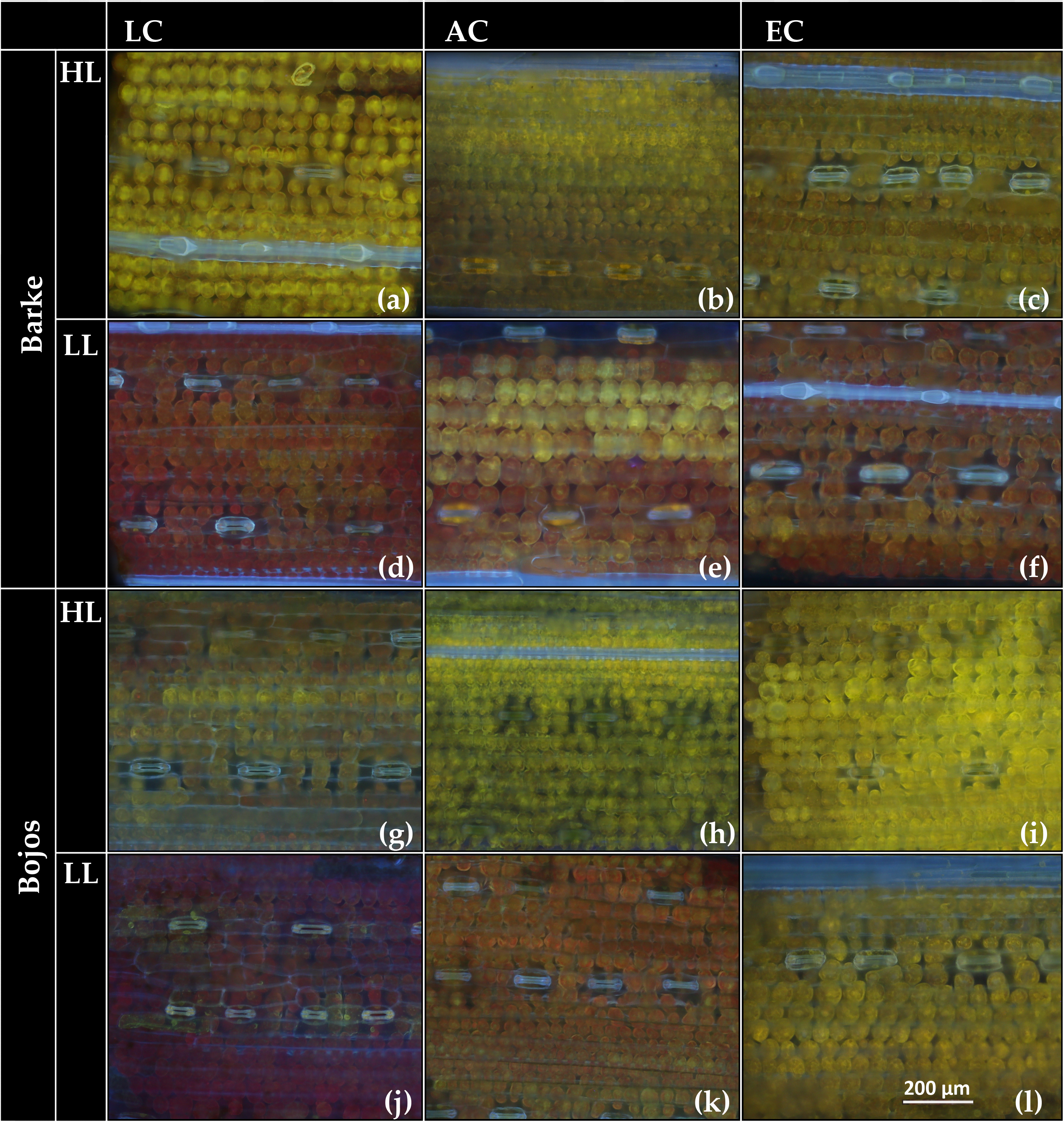

Supplement: Supplementary file 1 [file antioxidants-10-00385-s001.zip › Supplementary/Figure S2.tif]
